# Neuromodulatory Contribution to Muscle Force Production after Short-Term Unloading and Active Recovery

**DOI:** 10.1249/MSS.0000000000003473

**Published:** 2024-05-01

**Authors:** GIOVANNI MARTINO, GIACOMO VALLI, FABIO SARTO, MARTINO V. FRANCHI, MARCO V. NARICI, GIUSEPPE DE VITO

**Affiliations:** 1 Department of Biomedical Sciences, University of Padova, Padova, ITALY; 2 CIR-MYO Myology Centre, University of Padova, Padova, ITALY

**Keywords:** HIGH-DENSITY ELECTROMYOGRAPHY, MOTONEURON EXCITABILITY, MOTOR UNIT, MUSCLE DISUSE, PERSISTENT INWARD CURRENTS

## Abstract

**Purpose:**

Prior evidence has shown that neural factors contribute to the loss of muscle force after skeletal muscle disuse. However, little is known about the specific neural mechanisms altered by disuse. Persistent inward current (PIC) is an intrinsic property of motoneurons responsible for prolonging and amplifying the synaptic input, proportionally to the level of neuromodulation, thus influencing motoneuron discharge rate and force production. Here, we hypothesized that short-term unilateral lower limb suspension (ULLS) would reduce the neuromodulatory input associated with PIC, contributing to the reduction of force generation capacity. In addition, we tested whether physical exercise would restore the force generation capacity by reestablishing the initial level of neuromodulatory input.

**Methods:**

In 12 young adults, we assessed maximal voluntary contraction pre- and post-10 d of ULLS and after 21 d of active recovery (AR) based on resistance exercise. PIC was estimated from high-density surface electromyograms of the vastus lateralis muscle as the delta frequency (Δ*F*) of paired motor units calculated during isometric ramped contractions.

**Results:**

The values of Δ*F* were reduced after 10 d of ULLS (−33%, *P* < 0.001), but were fully reestablished after the AR (+29.4%, *P* < 0.001). The changes in estimated PIC values were correlated (*r* = 0.63, *P* = 0.004) with the reduction in maximal voluntary contraction after ULLS (−29%, *P* = 0.002) and its recovery after the AR (+28.5%, *P* = 0.003).

**Conclusions:**

Our findings suggest that PIC estimates are reduced by muscle disuse and may contribute to the loss of force production and its recovery with exercise. Overall, this is the first study demonstrating that, in addition to peripheral neuromuscular changes, central neuromodulation is a major contributor to the loss of force generation capacity after disuse, and can be recovered after resistance exercise.

Skeletal muscle disuse has a profound impact on the health of the neuromuscular system. The reduced mechanical loading and physical activity associated with muscle disuse can be a direct consequence of several conditions, such as musculoskeletal injuries, illness, aging, or prolonged exposure to microgravitational environments (1). In contrast, exercise countermeasures and restoration of physical activity represent the best nonpharmacological strategies for promoting neuromuscular system recovery after disuse (2). In this regard, resistance training is usually the first choice of rehabilitation to counteract the neuromuscular degeneration associated with a period of reduced muscle activity (3).

A primary consequence of disuse is a reduction in muscle force (4). Several experimental models, such as limb suspension or immobilization, dry immersion, step reduction, and bed rest, have been adopted to investigate the impact of muscle disuse (5–7). Prior reports investigating the mechanisms underpinning this disuse-induced muscle weakness have been primarily focused on the reduction in muscle mass (8), changes in tendon stiffness (9), and alterations in skeletal muscle fiber properties, including reduced oxidative capacity (10), impaired mechanical properties (2), and altered intracellular calcium handling (11). However, the amount of force produced cannot be explained only by peripheral muscle factors and greatly depends on motor units (MU) properties and their discharge rate (12). In a previous study, 48% of the loss in force was attributed to alterations in neural function and only 39% to muscular factors, whereas the remaining 13% was attributed to unexplained factors (13). In particular, the higher contribution among the neural variables was attributed to changes in central activation. In this regard, it is plausible to think that the neuromodulatory input to motoneurons may have an important yet uninvestigated role in muscle weakening after disuse.

Previous studies have shown a reduction in MU discharge rate after a period of disuse (14–19), suggesting a potential role of neural drive (the ensemble of motoneuron output (20)) as an early determinant of force loss (19). The changes in MU discharge rate are strongly influenced by the neuromodulatory contribution (21,22), associated with monoaminergic input from brainstem-derived neurotransmitters (e.g., serotonin and norepinephrine). These neurotransmitters act on G-proteins receptors altering the membrane potential and generating persistent inward currents (PIC). PIC constitute an intrinsic source of current, which prolong and amplify the excitatory synaptic input to motoneurons (23,24). Because the amplification of the synaptic input provides a powerful gain control mechanism over force generation (21), a reduction in the amplitude of PIC may contribute to the force loss after muscle disuse. In contrast, PIC amplitude has been shown to increase after resistance training (25) and may be associated with a recovery of muscle force capacity after disuse.

In our previous investigations (16,19), we found altered biomarkers of axonal damage and neuromuscular junction (NMJ) instability along with a decrease in the firing rates of low threshold MU after short-term disuse. However, the NMJ transmission remained largely unaltered, suggesting an early impairment upstream to the NMJ (central nervous system or motoneurons). Here, we aimed to investigate the potential role of central neuromodulation as an early determinant of force loss after disuse by assessing PIC. In humans, PIC can be indirectly estimated by measuring the differences in MU firing behavior during the ascending and descending phases of linear isometric ramp contractions (26,27). This technique is commonly referred as the paired MU analysis (28,29) and consists of calculating the delta frequency (Δ*F*) of a lower-threshold control unit at the time of recruitment and derecruitment of a higher-threshold test unit. Several studies have employed Δ*F* as an estimate of PIC in humans (26,30–34) and have shown its sensitivity in quantifying the neuromodulatory contribution to MU self-sustained firing (i.e., prolongation of the synaptic input) and its dependency on the monoaminergic drive (32,34–36). Estimates of PIC have been recently demonstrated to be reduced in older individuals both in upper and lower limbs (31,33,37) contributing to the loss of neuromuscular function with aging (38). However, whether muscle disuse is associated with a decrease in PIC estimates has yet to be determined.

In this study, we focused on quantifying the neuromodulatory contribution to neural drive and muscle force generation after short-term disuse and subsequent retraining. PIC were estimated from high-density surface electromyography (HDsEMG) recordings in the vastus lateralis (VL) muscle during isometric ramped contractions pre- and post-10 d of unilateral lower limb suspension (ULLS), and after 21 d of active recovery (AR). Values of bilateral maximal voluntary contraction (MVC) were also taken at each time point. We hypothesized that 10 d of ULLS would reduce MVC and PIC amplitude, and that 21 d of AR would restore the initial levels of neuromodulatory input and force capacity.

## MATERIALS AND METHODS

The experimental data analyzed in this study were part of a larger investigation designed to detect early neuromuscular changes after a short period of muscle unloading (16,19).

### Study Participants

Twelve recreationally active young male adults participated in this study (mean ± SD; age, 22.1 ± 2.9 yr; height, 1.78 ± 0.03 m; mass, 72.1 ± 7.1 kg). The inclusion criteria were individuals aged between 18 and 35 yr, with a body mass index ranging from 20 to 28 kg·m^−2^, and participation in recreational physical activities (one to three times a week, self-reported). Because females have a higher risk of venous thrombosis associated with ULLS (6), we only enrolled male individuals. None of the participants reported a history of neurological, circulatory, or neuromuscular disorders. All participants provided written informed consent before participation according to the protocols approved by the local Ethics Committee (HEC-DSB/01-18, Department of Biomedical Sciences, University of Padova, Italy).

### Experimental Design

**FIGURE 1:**
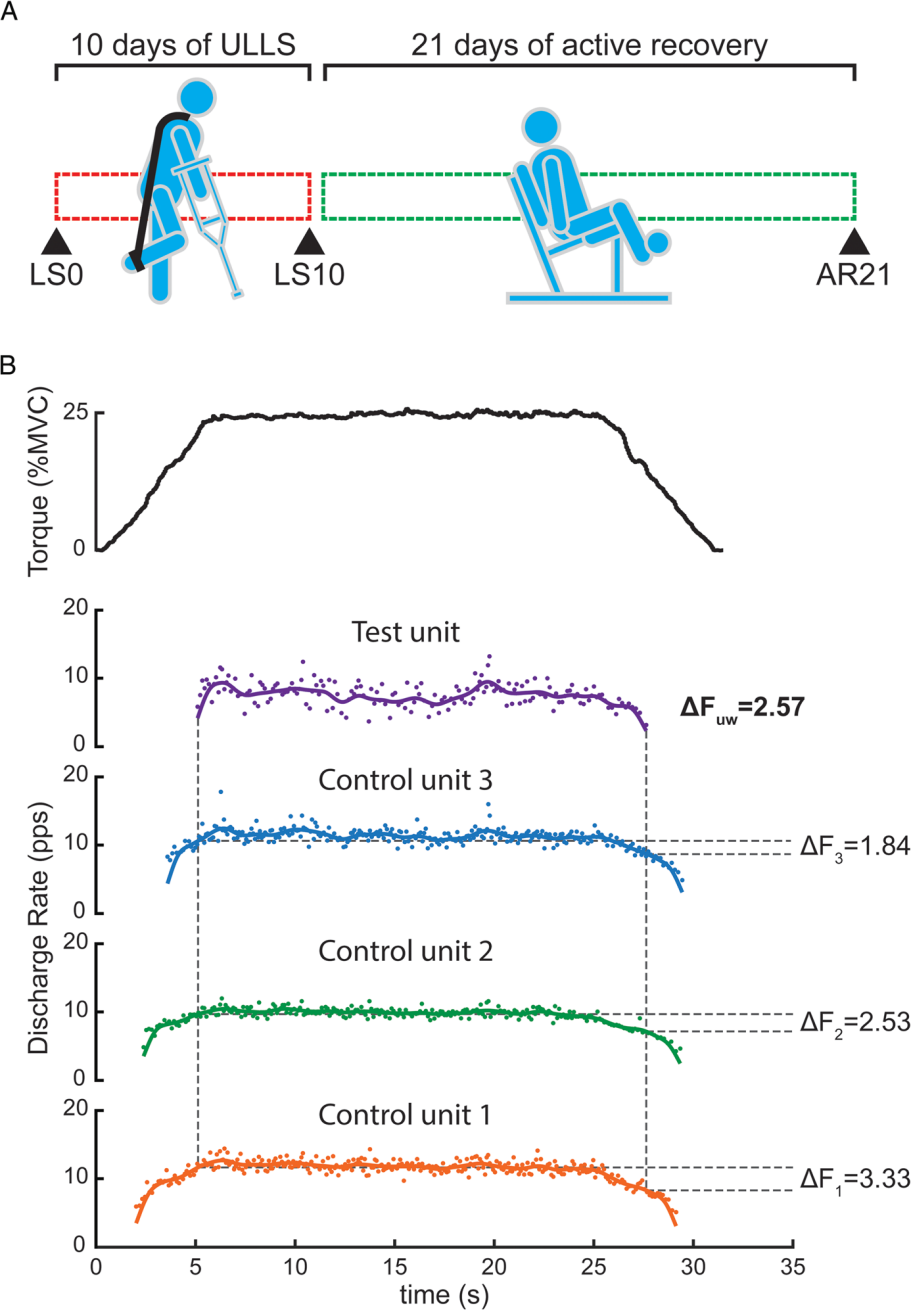
Study design (A) and example of delta frequency (Δ*F*) calculation using the paired MU analysis (B). (A) Experimental measurements were collected at baseline (day 0 before limb suspension, LS0), after 10 d of unilateral limb suspension (LS10), and after 21 d of AR (AR21). (B) Example of the torque profile from a single participant during isometric ramp contractions at 25% of maximum voluntary contraction (MVC, *top panel*). Subsequent panels show the smoothed instantaneous discharge rate of a test unit (*top trace*) and three control units. Each test-control unit pair was determined using the following criteria: the test unit was recruited at least 1 s after the control unit; the test unit was derecruited at least 1.5 s before the control unit; the control unit discharge rate modulation was >0.5 pps; the test unit was active continuously during the steady-state phase. Δ*F* was calculated as the difference in the smoothed instantaneous discharge rate (*horizontal dashed lines*) of each control unit at the time of recruitment and derecruitment (*vertical dashed lines*) of the test unit. The Δ*F* of each test-control unit pair was then averaged to obtain a unitwise Δ*F*_uw_ value per test unit.

Participants underwent a familiarization phase before the beginning of the study. During this phase, we explained the study procedures and instructed the participants on how to perform daily tasks while undergoing the ULLS (6). Experimental measurements were collected at baseline (day 0 before limb suspension, LS0), after 10 d of ULLS (LS10), and after 21 d of AR (AR21) (Fig. 1A). The duration of ULLS was based on our hypothesis that changes in neural drive could be early determinants of muscle force loss after disuse, and a marked strength loss was already shown after only 10 d of unilateral unloading (39). The duration of the AR period was determined by prior observations, indicating that achieving full muscle function recovery required a training period lasting twice the disuse phase (40). We also asked participants to abstain from intense activity, caffeine, and alcohol use during the 24 h preceding the data collection.

#### Unilateral lower limb suspension

We adopted the ULLS model originally described in Berg et al. (41). Briefly, participants wore a harness sling connecting the shoulder with the foot and suspending the leg in a slightly flexed position (15° to 20° of knee flexion) to prevent the dominant lower limb (right leg for all participants) from weight bearing. The shoe of the opposite leg was equipped with an elevated sole (50 mm) to avoid contact between the ground and the suspended leg. Participants used crutches to ambulate during the whole ULLS period, refraining from loading or actively contracting the suspended limb. Precautionary measures were also taken to prevent venous thromboembolism (16).

#### Active recovery

The AR phase consisted of a resistance training program starting ~72 h after the end of ULLS period. The training program comprised 3 sets of 10 repetitions of unilateral leg presses and leg extensions (both from ~0° to ~90° of knee flexion) at 70% of one repetition maximum (1 RM) performed 3 times per week (at least 24 h apart). Both concentric and eccentric phases of each single exercise lasted ~2 s, and sets were separated by 2-min rest. The 1 RM was estimated indirectly from the 4 to 6 RM and reassessed at the beginning of each training week (42).

### Experimental Protocol

Participants sat comfortably with their hips, knees, and ankles at 90° of flexion in the sagittal plane. The ankle of the dominant leg was secured with straps to a custom-made knee dynamometer incorporated with a load cell (RS 206-0290). The waist of each participant was secured with a strap to the seat to prevent compensatory movements during the trials. Each experimental session began with the assessment of the maximal voluntary isometric contraction (MVC) of the knee extensors muscles. After a warm-up period consisting of submaximal voluntary isometric contractions, participants were instructed to reach their maximum effort by pulling their dominant leg “as hard as possible” against the load cell and maintaining the contraction for 3 to 4 s. MVC was considered as the maximum tension value reached across three contractions separated by 1 min of rest.

After determining the MVC, participants performed two 30-s trapezoidal isometric contractions to 25% of MVC at a rate of torque development and decline of 5% MVC per second. Each trial was separated by 1 min of rest. A visual feedback with a trapezoidal template indicated the target force to be reached. For each trial, we collected the force signal from the load cell synchronized with the HDsEMG signal at 2048 Hz using a multichannel amplifier (Quattrocento; OTBioelettronica, Torino, Italy). The HDsEMG signal was collected from the VL muscle using a semidisposable 64-channel grid (13 × 5) with an 8-mm interelectrode distance (GR08MM1305, OTBioelettronica) and filled with a conductive cream (Ac cream, OTBioelettronica). After skin preparation (consisting of shaving, cleansing with 70% ethanol, and abrading), the grid was placed over the innervation zone of the muscle (43) and parallel to the muscle fascicle orientation (44). A low-intensity (8 to 16 mA) percutaneous electrical stimulation (Digitimer Ltd., Welwyn Garden, Hertfordshire, UK) was used to detect the innervation zone with the highest muscle twitch in the distal area of the muscle. Ultrasound imaging (Mylab70; Esaote, Genoa, Italy) was used to detect muscle fibers orientation. To ensure reproducibility in grid placement across data collection points, we marked the area of the skin surrounding the grid using a permanent marker.

### HDsEMG Analysis

After band-pass filtering (20 to 500 Hz, second-order Butterworth filter), the HDsEMG signal was decomposed into individual MU action potentials using the convolutive blind source separation technique (OTBioLab+; OTBioelettronica) (45). All decomposed MU spike trains were visually inspected and manually edited by an experienced investigator following previously published protocols (19), and only MU trains with a pulse-to-noise ratio (PNR) >28 dB were retained for further analysis (46). Because PIC are meant to prolong and amplify the synaptic input to motoneurons, for each identified MU, we computed the peak discharge rate and the total firing duration. In addition, we assessed the hysteresis property of PIC by computing the duration of the ascending and descending phases of the MU firing.

### Estimation of PIC

We estimated PIC amplitude using the paired MU analysis (26) and following the recommendations for standardized parameters described in Hassan et al. (30). The paired MU technique (Fig. 1B) consists of calculating the delta frequency (Δ*F*) of every possible combination pair of a lower threshold MU (control unit) at the time of recruitment and derecruitment of a higher threshold MU (test unit). First, we calculated the instantaneous MU discharge rate from the inverse of the interspike intervals. Then, the instantaneous discharge rate was smoothed using a 2-s Hanning window (31). The maximum value of the smoothed firing pattern was considered the peak discharge rate. The time from the first to the last firing event was considered the MU firing duration. Thus, low recruitment threshold MU (control units) were paired with higher recruitment threshold MU (test units) using the following criteria: 1) the test unit was recruited at least 1 s after the control unit; 2) the derecruitment of the control unit occurred at least 1.5 s after the derecruitment of the test unit; 3) the control unit discharge rate modulation was >0.5 pps; 4) the test unit was active continuously during the steady-state phase of the trapezoidal contraction. Finally, the delta frequency (Δ*F*) of each test-control unit pair was averaged to one per test unit (“unitwise” (31)). These criteria have previously been adopted to estimate PIC during trapezoidal contractions at intensities <30% of MVC (35).

### Statistical Analysis

All statistical tests were performed in R (version 4.1.0) using the RStudio environment (version 1.4.1717). Differences in MVC were investigated using one-way ANOVA and paired *t*-tests. Differences in Δ*F* and MU firing characteristics (peak discharge rate, firing duration, duration of the ascending and descending phases) across data collection points were assessed with separate linear mixed models using the lmerTest package. Each MU was treated as repeated measures, with the conditions (LS0, LS10, and AR21) as fixed factor and a random intercept for each participant. The estimated marginal mean at 95% confidence interval was computed using the emmeans package. Significance level was set at *α* = 0.05. Repeated-measures correlation (47) was used to quantify the within-individual association of paired measures averaged values at each condition (LS0, LS10, and AR21). Fixed slopes were used to estimate a single correlation coefficient for all participants. Correlation values were interpreted as moderate within 0.40 and 0.59, and strong within 0.60 and 0.79 (48).

## RESULTS

Of the 12 recruited participants, one withdrew from the study for personal reasons after baseline measures. Furthermore, for one participant, we were unable to identify MU with a PNR greater than 28 dB at LS10 and AR21. Another participant was excluded from the analysis because none of the identified MU at LS10 met the inclusion criteria required for the paired MU analysis. Hence, the final analysis included a total of 9 participants, 490 identified MU (183 at LS0, 159 at LS10, and 148 at AR21), and 237 test unit (99 at LS0, 63 at LS10, and 75 at AR21) along time.

### MVC torque

**FIGURE 2:**
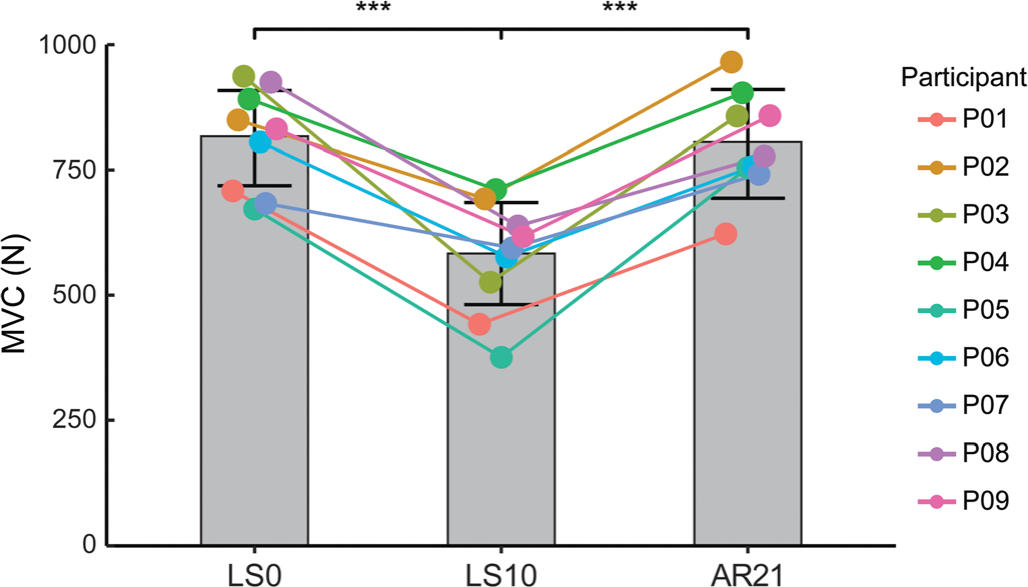
Bar plots illustrating the changes in maximum voluntary contraction (MVC) torque at baseline (day 0 before limb suspension, LS0), after 10 d of unilateral limb suspension (LS10), and after 21 d of AR (AR21). Data are displayed as mean ± SD values, and each *dot* refers to a single participant (n = 9). *Asterisks* denote significant values (***P < 0.001).

ULLS affected the maximal knee extensors torque. In all the participants, the MVC decreased at LS10 with respect to LS0 (−29%, *P* = 0.002, Fig. 2) and AR21 (−28.5%, *P* = 0.003, Fig. 2). MVC returned to the initial values of LS0 at AR21 (*P* = 0.99, Fig. 2).

### PIC estimates and MU firing characteristics

**FIGURE 3:**
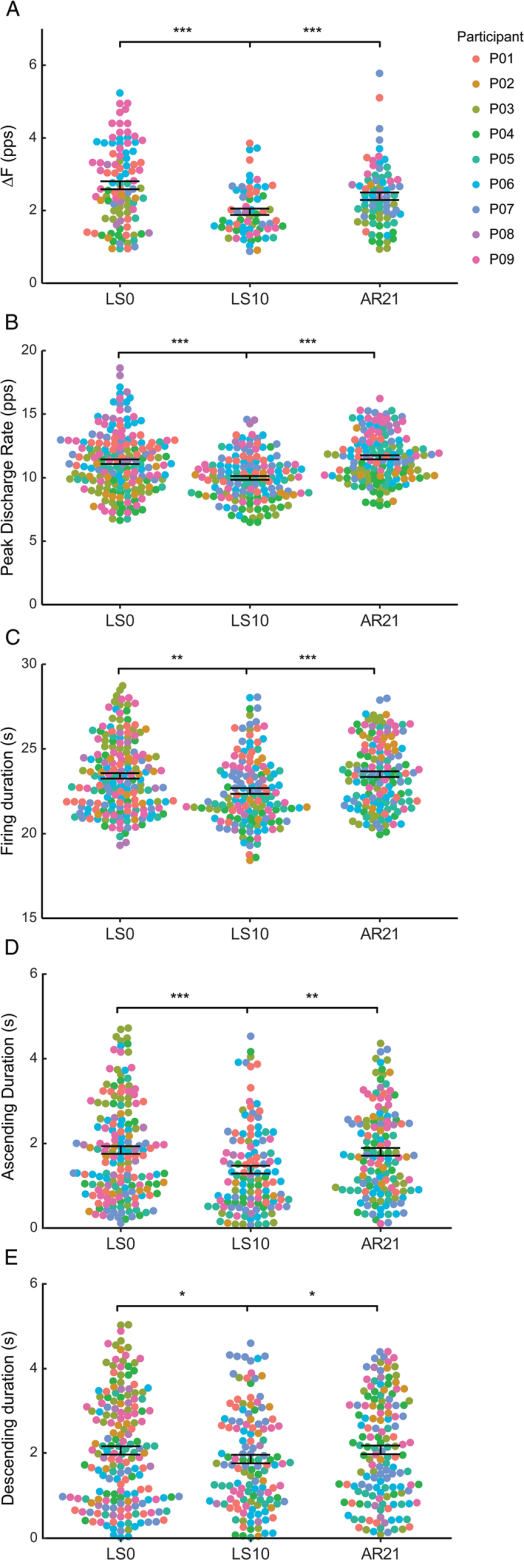
Swarm plots representing delta frequency (Δ*F*, A), peak discharge rate (B), firing duration (C), and durations of the ascending (D) and descending (E) phases of MU firings at baseline (day 0 before limb suspension, LS0), after 10 d of unilateral limb suspension (LS10), and after 21 d of AR (AR21). Data are displayed as mean ± SEM. Each *dot* refers to the averaged Δ*F* value per test unit (A, 99 at LS0, 63 at LS10, and 75 at AR21) or individual MU (B and C, 183 at LS0, 159 at LS10, and 148 at AR21) colored by participants. *Asterisks* denote significant differences (*P < 0.05, **P < 0.01, and ***P < 0.001).

**FIGURE 4:**
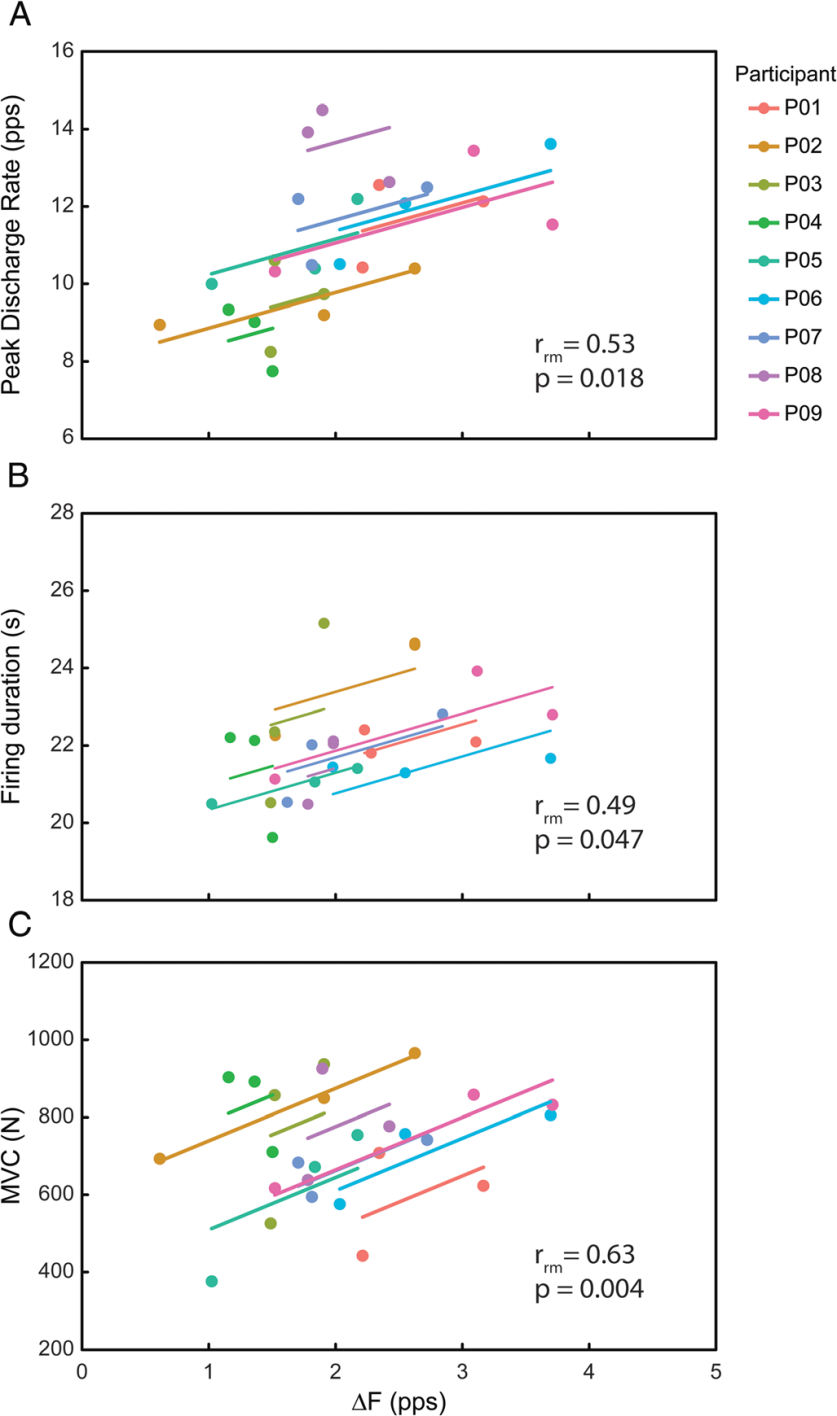
Repeated-measures correlation plots illustrating the association between delta frequency (Δ*F*) and peak discharge rate (A), firing duration (B), and MVC torque (C). Each parallel line fits the averaged data from an individual participant across data collection points colored by participant and with different shapes by condition. *r* and *P* values are reported in the lower right corner of each panel.

Linear mixed models showed an effect of ULLS and AR in PIC estimates and MU firing characteristics. Specifically, the unitwise delta frequency (Δ*F*) values significantly decreased from LS0 to LS10 (−33.2%, *P* < 0.001, Fig. 3A). At AR21, unitwise Δ*F* values significantly increased with respect to LS10 (+29.4%, *P* < 0.001, Fig. 3A) and were restored at LS0 values (*P* = 0.35, Fig. 3A). Similarly, peak discharge rate was significantly lower at LS10 compared with LS0 (−15.6%, *P* < 0.001, Fig. 3B) and AR21 (−17.6%, *P* < 0.001, Fig. 3B). No significant differences in peak discharge rate were found at AR21 with respect to LS0 (*P* = 0.59, Fig. 3B). Similarly, the MU firing duration decreased significantly at LS10 with respect to LS0 (−4.1%, *P* < 0.002, Fig. 3C). At AR21, the MU firing duration increased significantly with respect to LS10 (+3.2%, *P* < 0.001, Fig. 3C) and was restored to LS0 values (*P* = 0.62, Fig. 3C). The ascending phase of the MU firing was shorter at LS10 compared with LS0 (−41.9, *P* < 0.001, Fig. 3D) and AR21 (−47.9, *P* = 0.009, Fig. 3D), whereas no differences were found at AR21 with respect to LS0 (*P* = 0.85, Fig. 3D). Similarly, the descending phase of the MU firing was shorter at LS10 compared with LS0 (−19.1%, *P* = 0.018, Fig. 3E) and AR21 (−19.6%, *P* = 0.021, Fig. 3E) but not at AR21 compared with LS0 (*P* = 0.99, Fig. 3E). Repeated-measures correlations showed a positive association between the mean values of Δ*F* and the mean values of peak discharge rate, MU firing duration, and MVC across times. In particular, the changes in Δ*F* were moderately associated with the changes in peak discharge rate (*r* = 0.53, *P* = 0.018, Fig. 4A) and MU firing duration (*r* = 0.49, *P* = 0.047, Fig. 4B) and strongly associated with the changes in MVC (*r* = 0.63, *P* = 0.004, Fig. 4C).

## DISCUSSION

This is the first study assessing the contribution of PIC estimates (Δ*F*) to the loss in muscle force induced by a short period of muscle unloading in young healthy adults and its recovery after a resistance training program. Our results showed that, in the VL muscle, the amplitude of Δ*F* decreased after 10 d of ULLS, suggesting a reduction in neuromodulatory input to motoneurons with muscle disuse. Moreover, 21 d of AR restored the initial values of Δ*F*, further suggesting an increase in neuromodulatory input with resistance exercise. The observed changes in Δ*F* also correlated with changes in peak discharge rate and MU firing duration, consistently with an amplified and prolonged response of motoneurons proportionally to the level of neuromodulatory input. Overall, our findings suggest that changes in neuromodulatory input may contribute to the loss of force production capacity after disuse and its recovery with resistance-based exercise.

The notion that the loss of muscle strength after muscle disuse is largely due to neural factors has been postulated by several authors (41,49). In particular, previous studies have attributed most of the decrease in muscle strength to changes in central activation (50–52). Our findings suggest that the reduction in PIC estimates contributed to the loss of force production after muscle disuse, negatively regulating the MU firing rates, as previously shown in this cohort (19). PIC plays a major role in the enhancement of the synaptic input to motoneurons and is dependent on the neuromodulatory input associated with monoaminergic drive (24). Here we found that at the same relative levels of muscle force, Δ*F* decreased by about 30% after ULLS, suggesting that a decrease in neuromodulatory input may have contributed to the reduction of the MVC (*r* = 0.63). Evidence of the influence of neuromodulation on maximal force production has also been previously reported both in experimental data (53,54) and on simulations (55). These studies have suggested that, in absence of neuromodulatory input, the ionotropic inputs alone could not provide sufficient drive to reach the maximum force output, whereas increasing the concentrations of serotonin improves the ability to sustain higher MVC.

Decreased values of Δ*F* after ULLS suggest a lower monoaminergic drive onto motoneurons with disuse. Consistently with our results, a previous modeling study (56) showed a reduction of persistent Na + currents in mice that underwent 8 wk of hindlimb unloading, a well-established animal model of disuse. Similarly, 2 wk of hindlimb unloading reduced the excitability of motoneurons in rats (57). Further evidence of reduced monoaminergic drive with disuse comes from a leg immobilization study in humans (58). After 5 d of leg immobilization, the plasma concentration level of tryptophan increased, suggesting a decrease in serotonin synthesis with muscle disuse. Indeed, despite that several metabotropic receptors can be responsible for neuromodulation (59,60), many studies have focused their attention on the primary role of serotonin in generating PIC (35,61,62). Tryptophan is the only precursor involved in the synthesis of serotonin, and serotonin release has been shown to vary readily with alteration in tryptophan availability (63,64). Thus, an increase in tryptophan availability with muscle disuse could be associated with a decrease in serotoninergic drive and, consequently, with a decrease in PIC. However, no studies have directly related the plasma concentration levels of tryptophan or other markers of serotonergic activity to PIC estimates.

Alternatively, the decrease in Δ*F* values might be related to an increase in central inhibition. Indeed, a concurrent mechanism of central modulation to PIC is represented by the extent and pattern of inhibition to excitation. Experiments on decerebrated cats have shown a reduction in PIC to be proportional to the magnitude of an inhibition stimuli applied nerve afferents of antagonist muscle (65). A reduction in PIC with reciprocal inhibition induced by vibration or electrical stimulation of antagonist muscles has also been shown in humans (32,34). However, several studies on disuse provided evidence of an increase in H-reflex amplitude (13,50,66), supporting a reduction rather than an increase of presynaptic inhibition after muscle disuse.

The effect of PIC on muscle activation was also evident from the correlation between peak discharge rate and MU firing duration. Indeed, PIC modulates the motoneuron discharge rate by amplifying the synaptic input (36,55). The correlation (*r* = 0.53) between the changes in Δ*F* and peak discharge rate across time further confirms this association and the influence of disuse on PIC. In addition, PIC are known to prolong the sustained MU firing (23). We found a moderate correlation (*r* = 0.49) between the changes in Δ*F* and MU firing duration across time. The Δ*F* scores are an estimation of contribution of PIC to MU firing rate hysteresis (26). In this regard, we also found a reduction in the duration of the ascending and descending phases of MU firing. Furthermore, the reduction in PIC estimates is consistent with the alterations in absolute MU recruitment and derecruitment thresholds previously reported in this cohort (18). In addition, the moderate values of correlation between Δ*F* and peak discharge rate and MU firing duration suggest that other factors, such as other alterations in the biophysical properties of the motoneurons, may contribute to the observed changes in MU firing rates. However, this is the first study demonstrating the contribution of PIC to altered MU discharge rates after disuse.

Twenty-one days of resistance training restored the baseline values of Δ*F*. The effect of physical activity on the monoaminergic system is well known. Increases in PIC estimates have previously been reported in soleus muscle after 6 wk of high-intensity resistance training in older adults (25). Higher discharge rate during the plateau phase of submaximal ramp contraction was observed in tibialis anterior muscle after 4 wk of strength training in young adults (67). Several other studies have shown that resistance training is effective in promoting neuromodulatory activity and increasing motoneuron excitability in both animals and humans (68,69). However, this is the first study investigating the changes in neuromodulatory drive after resistance training as a countermeasure to short-term unloading. A period of 21 d of resistance training was sufficient to reestablish the baseline values of Δ*F*. Based on our results, we could speculate that the increase in Δ*F* at AR21 was associated with a recovery in monoaminergic drive after resistance exercise and essential for higher force generation.

### Limitations

The analysis proposed in this study was limited to the total amount of MU that we were able to decompose from VL at 25% of MVC. Thus, our interpretation may not apply to MU recruited at higher forces and/or in different muscles. However, forces close to 25% of MVC are extensively used for Δ*F* calculation (32,34) and are functionally relevant to common daily activities such as walking (70). Furthermore, ramps were performed at relative rather than absolute values of muscle torque. Because the monoaminergic drive increases proportionally to the level of voluntary drive (33), the reduction of Δ*F* after ULLS may be a consequence of the reduced contraction intensity rather than a contributor to the total muscle force generation capacity. However, we think that the use of the same absolute torque values would have overestimated the maximal contractile capacity after ULLS, because many neural and muscle factors concur with the loss of muscle force (4,13). Therefore, similarly to other longitudinal studies on MU (35,67), the changes in Δ*F* were assessed at the same relative levels of muscle torque. In addition, we used 30-s trapezoidal contractions in this study. Despite that the estimation of PIC has been successfully performed on long contractions (35,71), another mechanism known as spike-frequency adaptation could be altered by the level of physical activity (72,73) and may contribute to the changes in PIC estimates with muscle disuse (27). Finally, we did not include a rate–rate correlation threshold among our criteria for MU pairing. This threshold ensures the physiological assumption for PIC estimation that paired MU receive common synaptic input (26), but it dramatically decreases the number of MU pairs. However, a previous work showed that the removal of rate–rate correlation threshold did not affect Δ*F* values or variance (30). Also, estimating PIC only requires the reporter unit to reflect the relative synaptic drive of the test unit during recruitment and derecruitment (29), whereas the rates might vary independently over prolonged contractions as in our protocol. However, further studies are needed to address these limitations.

## CONCLUSIONS

It is well known that muscle force loss during disuse and recovery after exercise countermeasures are influenced by changes in structural components (such as muscle mass, tendon stiffness, fibers properties) and neural factors (NMJ transmission, oxidative capacity, intracellular calcium handling), which are mostly regulating muscle force production at the local/peripheral level. Here we demonstrated that, at a central level, PIC could be one of the factors contributing to muscle force loss and recovery after lower limb unloading and reloading. In particular, we found that the amplitude of PIC estimates in VL was reduced by ULLS, contributing to the loss of force generation capacity. We also found that 21 d of AR were sufficient to restore the initial values of PIC estimates, highlighting the importance of exercise in reactivating the neuromodulatory system. Understanding the role of neuromodulation as a contributor to muscle force tuning with disuse may have important implications for the preservation/recovery of muscle functionality in several conditions such as prolonged limb unloading due to musculoskeletal injuries or diseases, or as a consequence of space flight in astronauts.

## References

[1] Nunes EA Stokes T McKendry J Currier BS Phillips SM. Disuse-induced skeletal muscle atrophy in disease and nondisease states in humans: mechanisms, prevention, and recovery strategies. *Am J Physiol Cell Physiol*. 2022;322(6):C1068–84. 35476500 10.1152/ajpcell.00425.2021

[2] Brocca L Longa E Cannavino J, . Human skeletal muscle fibre contractile properties and proteomic profile: adaptations to 3 weeks of unilateral lower limb suspension and active recovery. *J Physiol*. 2015;593(24):5361–85. 26369674 10.1113/JP271188 PMC4704515

[3] Bickel CS Cross JM Bamman MM. Exercise dosing to retain resistance training adaptations in young and older adults. *Med Sci Sports Exerc*. 2011;43(7):1177–87. 21131862 10.1249/MSS.0b013e318207c15d

[4] Clark BC Fernhall B Ploutz-Snyder LL. Adaptations in human neuromuscular function following prolonged unweighting: I. Skeletal muscle contractile properties and applied ischemia efficacy. *J Appl Physiol (1985)*. 2006;101(1):256–63. 16514004 10.1152/japplphysiol.01402.2005

[5] Pavy-Le Traon A Heer M Narici MV Rittweger J Vernikos J. From space to Earth: advances in human physiology from 20 years of bed rest studies (1986–2006). *Eur J Appl Physiol*. 2007;101(2):143–94. 17661073 10.1007/s00421-007-0474-z

[6] Tesch PA Lundberg TR Fernandez-Gonzalo R. Unilateral lower limb suspension: From subject selection to “omic” responses. *J Appl Physiol (1985)*. 2016;120(10):1207–14. 26846557 10.1152/japplphysiol.01052.2015

[7] Tomilovskaya E Shigueva T Sayenko D Rukavishnikov I Kozlovskaya I. Dry immersion as a ground-based model of microgravity physiological effects. *Front Physiol*. 2019;10:284. 30971938 10.3389/fphys.2019.00284 PMC6446883

[8] Bell KE von Allmen MT Devries MC Phillips SM. Muscle disuse as a pivotal problem in sarcopenia-related muscle loss and dysfunction. *J Frailty Aging*. 2016;5(1):33–41. 26980367 10.14283/jfa.2016.78

[9] de Boer MD Maganaris CN Seynnes OR Rennie MJ Narici MV. Time course of muscular, neural and tendinous adaptations to 23 day unilateral lower-limb suspension in young men. *J Physiol*. 2007;583(Pt 3):1079–91. 17656438 10.1113/jphysiol.2007.135392 PMC2277190

[10] Mirzoev TM Sharlo KA Shenkman BS. The role of GSK-3β in the regulation of protein turnover, myosin phenotype, and oxidative capacity in skeletal muscle under disuse conditions. *Int J Mol Sci*. 2021;22(10):5081. 34064895 10.3390/ijms22105081 PMC8151958

[11] Monti E Reggiani C Franchi MV, . Neuromuscular junction instability and altered intracellular calcium handling as early determinants of force loss during unloading in humans. *J Physiol*. 2021;599(12):3037–61. 33881176 10.1113/JP281365 PMC8359852

[12] Enoka RM Duchateau J. Rate coding and the control of muscle force. *Cold Spring Harb Perspect Med*. 2017;7(10):a029702. 28348173 10.1101/cshperspect.a029702 PMC5629984

[13] Clark BC Manini TM Bolanowski SJ Ploutz-Snyder LL. Adaptations in human neuromuscular function following prolonged unweighting: II. Neurological properties and motor imagery efficacy. *J Appl Physiol (1985)*. 2006;101(1):264–72. 16514003 10.1152/japplphysiol.01404.2005

[14] Duchateau J Hainaut K. Effects of immobilization on contractile properties, recruitment and firing rates of human motor units. *J Physiol*. 1990;422:55–65. 2352193 10.1113/jphysiol.1990.sp017972 PMC1190120

[15] Inns TB Bass JJ Hardy EJO, . Motor unit dysregulation following 15 days of unilateral lower limb immobilisation. *J Physiol*. 2022;600(21):4753–69. 36088611 10.1113/JP283425 PMC9827843

[16] Sarto F Stashuk DW Franchi MV, . Effects of short-term unloading and active recovery on human motor unit properties, neuromuscular junction transmission and transcriptomic profile. *J Physiol*. 2022;600(21):4731–51. 36071599 10.1113/JP283381 PMC9828768

[17] Seki K Taniguchi Y Narusawa M. Effects of joint immobilization on firing rate modulation of human motor units. *J Physiol*. 2001;530(Pt 3):507–19. 11158280 10.1111/j.1469-7793.2001.0507k.x PMC2278422

[18] Seki K Kizuka T Yamada H. Reduction in maximal firing rate of motoneurons after 1-week immobilization of finger muscle in human subjects. *J Electromyogr Kinesiol*. 2007;17(2):113–20. 16448820 10.1016/j.jelekin.2005.10.008

[19] Valli G Sarto F Casolo A, . Lower limb suspension induces threshold-specific alterations of motor units properties that are reversed by active recovery. *J Sport Health Sci*. 2024;13(2):264–76. 37331508 10.1016/j.jshs.2023.06.004 PMC10980901

[20] Farina D Negro F Muceli S Enoka RM. Principles of motor unit physiology evolve with advances in technology. *Physiology (Bethesda)*. 2016;31(2):83–94. 26889014 10.1152/physiol.00040.2015

[21] Heckman CJ Mottram C Quinlan K Theiss R Schuster J. Motoneuron excitability: the importance of neuromodulatory inputs. *Clin Neurophysiol*. 2009;120(12):2040–54. 19783207 10.1016/j.clinph.2009.08.009 PMC7312725

[22] Kavanagh JJ Taylor JL. Voluntary activation of muscle in humans: does serotonergic neuromodulation matter? *J Physiol*. 2022;600(16):3657–70. 35864781 10.1113/JP282565 PMC9541597

[23] Binder MD Powers RK Heckman CJ. Nonlinear input-output functions of motoneurons. *Physiology (Bethesda)*. 2020;35(1):31–9. 31799904 10.1152/physiol.00026.2019 PMC7132324

[24] Heckman CJ Gorassini MA Bennett DJ. Persistent inward currents in motoneuron dendrites: implications for motor output. *Muscle Nerve*. 2005;31(2):135–56. 15736297 10.1002/mus.20261

[25] Orssatto LBR Rodrigues P Mackay K, . Intrinsic motor neuron excitability is increased after resistance training in older adults. *J Neurophysiol*. 2023;129(3):635–50. 36752407 10.1152/jn.00462.2022

[26] Gorassini M Yang JF Siu M Bennett DJ. Intrinsic activation of human motoneurons: possible contribution to motor unit excitation. *J Neurophysiol*. 2002;87(4):1850–8. 11929906 10.1152/jn.00024.2001

[27] Vandenberk MS Kalmar JM. An evaluation of paired motor unit estimates of persistent inward current in human motoneurons. *J Neurophysiol*. 2014;111(9):1877–84. 24523524 10.1152/jn.00469.2013

[28] Gorassini MA Knash ME Harvey PJ Bennett DJ Yang JF. Role of motoneurons in the generation of muscle spasms after spinal cord injury. *Brain*. 2004;127(Pt 10):2247–58. 15342360 10.1093/brain/awh243

[29] Powers RK Nardelli P Cope TC. Estimation of the contribution of intrinsic currents to motoneuron firing based on paired motoneuron discharge records in the decerebrate cat. *J Neurophysiol*. 2008;100(1):292–303. 18463182 10.1152/jn.90296.2008 PMC2493492

[30] Hassan A Thompson CK Negro F, . Impact of parameter selection on estimates of motoneuron excitability using paired motor unit analysis. *J Neural Eng*. 2020;17(1):016063. 31801123 10.1088/1741-2552/ab5eda PMC7295184

[31] Hassan AS Fajardo ME Cummings M, . Estimates of persistent inward currents are reduced in upper limb motor units of older adults. *J Physiol*. 2021;599(21):4865–82. 34505294 10.1113/JP282063 PMC8560565

[32] Mesquita RNO Taylor JL Trajano GS, . Effects of reciprocal inhibition and whole-body relaxation on persistent inward currents estimated by two different methods. *J Physiol*. 2022;600(11):2765–87. 35436349 10.1113/JP282765 PMC9325475

[33] Orssatto LBR Borg DN Blazevich AJ Sakugawa RL Shield AJ Trajano GS. Intrinsic motoneuron excitability is reduced in soleus and tibialis anterior of older adults. *Geroscience*. 2021;43(6):2719–35. 34716899 10.1007/s11357-021-00478-z PMC8556797

[34] Orssatto LBR Fernandes GL Blazevich AJ Trajano GS. Facilitation-inhibition control of motor neuronal persistent inward currents in young and older adults. *J Physiol*. 2022;600(23):5101–17. 36284446 10.1113/JP283708 PMC10092053

[35] Goodlich BI Del Vecchio A Horan SA Kavanagh JJ. Blockade of 5-HT2 receptors suppresses motor unit firing and estimates of persistent inward currents during voluntary muscle contraction in humans. *J Physiol*. 2023;601(6):1121–38. 36790076 10.1113/JP284164

[36] Mackay Phillips K Orssatto LBR Polman R Van der Pols JC Trajano GS. The effects of α-lactalbumin supplementation and handgrip contraction on soleus motoneuron excitability. *Eur J Appl Physiol*. 2023;123(2):395–404. 36443491 10.1007/s00421-022-05101-3

[37] Guo Y Jones E Škarabot J, . Common synaptic inputs and persistent inward currents of vastus lateralis motor units are reduced in older male adults. *Geroscience*. 2024;46(3):3249–61. 38238546 10.1007/s11357-024-01063-w PMC11009172

[38] Orssatto LBR Blazevich AJ Trajano GS. Ageing reduces persistent inward current contribution to motor neurone firing: potential mechanisms and the role of exercise. *J Physiol*. 2023;601(17):3705–16. 37488952 10.1113/JP284603

[39] Berg HE Tesch PA. Changes in muscle function in response to 10 days of lower limb unloading in humans. *Acta Physiol Scand*. 1996;157(1):63–70. 8735655 10.1046/j.1365-201X.1996.476217000.x

[40] Suetta C Hvid LG Justesen L, . Effects of aging on human skeletal muscle after immobilization and retraining. *J Appl Physiol (1985)*. 2009;107(4):1172–80. 19661454 10.1152/japplphysiol.00290.2009

[41] Berg HE Dudley GA Häggmark T Ohlsén H Tesch PA. Effects of lower limb unloading on skeletal muscle mass and function in humans. *J Appl Physiol (1985)*. 1991;70(4):1882–5. 2055867 10.1152/jappl.1991.70.4.1882

[42] Brzycki M. Strength testing—predicting a one-rep max from reps-to-fatigue. *J Phys Educ Recreat Dance*. 1993;64(1):88–90.

[43] Botter A Oprandi G Lanfranco F Allasia S Maffiuletti NA Minetto MA. Atlas of the muscle motor points for the lower limb: implications for electrical stimulation procedures and electrode positioning. *Eur J Appl Physiol*. 2011;111(10):2461–71. 21796408 10.1007/s00421-011-2093-y

[44] Hug F Del Vecchio A Avrillon S Farina D Tucker K. Muscles from the same muscle group do not necessarily share common drive: evidence from the human triceps surae. *J Appl Physiol (1985)*. 2021;130(2):342–54. 33242301 10.1152/japplphysiol.00635.2020

[45] Negro F Muceli S Castronovo AM Holobar A Farina D. Multi-channel intramuscular and surface EMG decomposition by convolutive blind source separation. *J Neural Eng*. 2016;13(2):026027. 26924829 10.1088/1741-2560/13/2/026027

[46] Holobar A Minetto MA Farina D. Accurate identification of motor unit discharge patterns from high-density surface EMG and validation with a novel signal-based performance metric. *J Neural Eng*. 2014;11(1):016008. 24654270 10.1088/1741-2560/11/1/016008

[47] Bakdash JZ Marusich LR. Repeated measures correlation. *Front Psychol*. 2017;8:456. 28439244 10.3389/fpsyg.2017.00456 PMC5383908

[48] Evans JD. *Straightforward statistics for the behavioral sciences*. Belmont, CA, US: Thomson Brooks/Cole Publishing Co; 1996. xxii, 600 p.

[49] Deschenes MR Giles JA McCoy RW Volek JS Gomez AL Kraemer WJ. Neural factors account for strength decrements observed after short-term muscle unloading. *Am J Physiol Regul Integr Comp Physiol*. 2002;282(2):R578–83. 11792669 10.1152/ajpregu.00386.2001

[50] Duchateau J. Bed rest induces neural and contractile adaptations in triceps surae. *Med Sci Sports Exerc*. 1995;27(12):1581–9. 8614311

[51] Kawakami Y Akima H Kubo K, . Changes in muscle size, architecture, and neural activation after 20 days of bed rest with and without resistance exercise. *Eur J Appl Physiol*. 2001;84(1–2):7–12. 11394257 10.1007/s004210000330

[52] Sale DG McComas AJ MacDougall JD Upton AR. Neuromuscular adaptation in human thenar muscles following strength training and immobilization. *J Appl Physiol Respir Environ Exerc Physiol*. 1982;53(2):419–24. 6288637 10.1152/jappl.1982.53.2.419

[53] Binder MD. Intrinsic dendritic currents make a major contribution to the control of motoneurone discharge. *J Physiol*. 2003;552(Pt 3):665. 14514880 10.1113/jphysiol.2003.054817 PMC2343444

[54] Kavanagh JJ McFarland AJ Taylor JL. Enhanced availability of serotonin increases activation of unfatigued muscle but exacerbates central fatigue during prolonged sustained contractions. *J Physiol*. 2019;597(1):319–32. 30328105 10.1113/JP277148 PMC6312415

[55] Heckman CJ Lee RH Brownstone RM. Hyperexcitable dendrites in motoneurons and their neuromodulatory control during motor behavior. *Trends Neurosci*. 2003;26(12):688–95. 14624854 10.1016/j.tins.2003.10.002

[56] Banzrai C Nodera H Kawarai T, . Impaired axonal Na + current by hindlimb unloading: implication for disuse neuromuscular atrophy. *Front Physiol*. 2016;7:36. 26909041 10.3389/fphys.2016.00036 PMC4754663

[57] Cormery B Beaumont E Csukly K Gardiner P. Hindlimb unweighting for 2 weeks alters physiological properties of rat hindlimb motoneurones. *J Physiol*. 2005;568(Pt 3):841–50. 16123107 10.1113/jphysiol.2005.091835 PMC1464183

[58] Dirks ML Wall BT Nilwik R Weerts DHJM Verdijk LB Van Loon LJC. Skeletal muscle disuse atrophy is not attenuated by dietary protein supplementation in healthy older men. *J Nutr*. 2014;144(8):1196–203. 24919692 10.3945/jn.114.194217

[59] Fyffe R. Spinal motoneurons: synaptic inputs and receptor organization. *Motor Neurobiol Spinal Cord*. 2001;21–46.

[60] Svirskis G Hounsgaard J. Transmitter regulation of plateau properties in turtle motoneurons. *J Neurophysiol*. 1998;79(1):45–50. 9425175 10.1152/jn.1998.79.1.45

[61] Hounsgaard J Kiehn O. Serotonin-induced bistability of turtle motoneurones caused by a nifedipine-sensitive calcium plateau potential. *J Physiol*. 1989;414:265–82. 2607432 10.1113/jphysiol.1989.sp017687 PMC1189141

[62] Perrier J-F Rasmussen HB Christensen RK Petersen AV. Modulation of the intrinsic properties of motoneurons by serotonin. *Curr Pharm Des*. 2013;19(24):4371–84. 23360270 10.2174/13816128113199990341

[63] Fernstrom JD. Role of precursor availability in control of monoamine biosynthesis in brain. *Physiol Rev*. 1983;63(2):484–546. 6132421 10.1152/physrev.1983.63.2.484

[64] Schaechter JD Wurtman RJ. Serotonin release varies with brain tryptophan levels. *Brain Res*. 1990;532(1–2):203–10. 1704290 10.1016/0006-8993(90)91761-5

[65] Kuo JJ Lee RH Johnson MD Heckman HM Heckman CJ. Active dendritic integration of inhibitory synaptic inputs in vivo. *J Neurophysiol*. 2003;90(6):3617–24. 12944534 10.1152/jn.00521.2003

[66] Lundbye-Jensen J Nielsen JB. Immobilization induces changes in presynaptic control of group Ia afferents in healthy humans. *J Physiol*. 2008;586(17):4121–35. 18599534 10.1113/jphysiol.2008.156547 PMC2652189

[67] Del Vecchio A Casolo A Negro F, . The increase in muscle force after 4 weeks of strength training is mediated by adaptations in motor unit recruitment and rate coding. *J Physiol*. 2019;597(7):1873–87. 30727028 10.1113/JP277250 PMC6441907

[68] Christie A Kamen G. Short-term training adaptations in maximal motor unit firing rates and after hyperpolarization duration. *Muscle Nerve*. 2010;41(5):651–60. 19941348 10.1002/mus.21539

[69] Pietta-Dias C Bello MD da Silva R, . Differential impact of endurance, strength, or combined training on quality of life and plasma serotonin in healthy older women. *Aging Clin Exp Res*. 2019;31(11):1573–81. 30656562 10.1007/s40520-019-01120-x

[70] Bohm S Marzilger R Mersmann F Santuz A Arampatzis A. Operating length and velocity of human vastus lateralis muscle during walking and running. *Sci Rep*. 2018;8(1):5066. 29567999 10.1038/s41598-018-23376-5 PMC5864755

[71] Orssatto LBR Mackay K Shield AJ Sakugawa RL Blazevich AJ Trajano GS. Estimates of persistent inward currents increase with the level of voluntary drive in low-threshold motor units of plantar flexor muscles. *J Neurophysiol*. 2021;125(5):1746–54. 33788617 10.1152/jn.00697.2020

[72] MacDonell CW Button DC Beaumont E Cormery B Gardiner PF. Plasticity of rat motoneuron rhythmic firing properties with varying levels of afferent and descending inputs. *J Neurophysiol*. 2012;107(1):265–72. 21957225 10.1152/jn.00122.2011

[73] Revill AL Fuglevand AJ. Effects of persistent inward currents, accommodation, and adaptation on motor unit behavior: a simulation study. *J Neurophysiol*. 2011;106(3):1467–79. 21697447 10.1152/jn.00419.2011 PMC3174816

